# Evaluation of the Nature and Concentration of the Surfactant on the Properties of Red Mud/Metakaolin Porous Geopolymers Foamed with Aluminium

**DOI:** 10.3390/ma15217486

**Published:** 2022-10-25

**Authors:** Senem Bilici, João Carvalheiras, João A. Labrincha, Rui M. Novais

**Affiliations:** 1 Department of Materials and Ceramic Engineering, CICECO-Aveiro Institute of Materials, University of Aveiro, Campus Universitário de Santiago, 3810-193 Aveiro, Portugal; 2 Department of Civil Engineering, Yildiz Technical University, 34220 Istanbul, Türkiye; 3 Department of Construction Technology, Istanbul Aydin University, 34295 Istanbul, Türkiye

**Keywords:** alkali-activated material, red mud, pore size distribution, water absorption, thermal conductivity

## Abstract

The chemical foaming technique is possibly the most common method of producing porous geopolymers. Despite this, to date, the role of the content and type of surfactant on the pore size distribution of porous geopolymers is not fully perceived, as constant surfactant dosages are usually employed. In addition, the comparison of literature studies is challenging since a distinct mixture of designs is employed. This investigation intends to provide additional insights on the topic, focusing on synthesizing red mud/metakaolin geopolymer foams and envisioning their use in thermal insulating applications. Various mixtures were prepared using three commercially available surfactants, namely Hostapur OSB, sodium dodecyl sulfate (SDS), and Triton X114. The content of the surfactant (0.025, 0.05, and 0.075 wt.%) and the amount of the foaming agent (aluminum powder, Al; 0.05, 0.075, and 0.10 wt.%) was modified, keeping the binder composition constant and the physical properties of the produced geopolymers were characterized. Results show that the combination between sodium dodecyl sulfate (0.025 wt.%) and aluminum (0.10 wt.%) leads to the strongest reduction in the foam density, the lowest value here reported being −400 kg/m^3^. On the other hand, samples produced with Hostapur OSB have much higher open porosity (up to 47.7%) and water absorption (up to 80.4%) values, showing that this surfactant leads to a pore network with higher connectivity. In addition, the microstructure of the foams, particularly pore morphology (size and shape) and connectivity between the produced pores are highly dependent on the type of surfactant, sodium dodecyl sulfate generating coarser pore size distribution with round, but mostly closed pores, while a narrower pore size distribution coupled with smaller size pores is seen with the Hostapur. These results suggest the feasibility of tuning the foams’ properties (porosity and mechanical performance) according to the application by the proper combination of the type of surfactant and their concentration, enabling their use as thermal and acoustic insulators or as filters/membranes in wastewater treatment systems.

## 1. Introduction

Energy consumption has been increasing since 2000, associated with the escalation of the world population and the need to use energy for human subsistence. In line with this, a recent report (BP Statistical Review of World Energy) shows that the energy used in buildings is increasing. The European Union has been one of the active voices in energy change and intervention to promote energy efficiency at a global level. The very high values of worldwide energy consumption have a major impact on the production of greenhouse gases. A promising strategy to tackle energy consumption is to increase the energy efficiency in buildings since this sector accounts for 36% of the global final energy needs [1]. An interesting option can be using lightweight and low thermal conductivity materials, as they can mitigate energy losses in buildings and reduce the use of heating, ventilation, and cooling systems. The use of lightweight geopolymers is particularly promising [2]. These materials can be produced at much lower temperatures (below 100 °C) compared to Portland cement (PC) [3] and are widely considered environmentally friendly. Another important aspect is that PC produces a huge amount of greenhouse gases (CO_2_) during the calcination of clinker. Geopolymers are produced from an aluminosilicate source combined with an alkali agent (usually NaOH or KOH combined with silicates) [4]. Geopolymers are formed in a three-step reaction that can be simplified as: (1) dissolution-coagulation; (2) coagulation-condensation; (3) condensation-crystallization [5]. This novel binder can be produced using a wide range of aluminosilicate raw materials, including metakaolin (MK), but wastes, e.g., fly ash (FA), glass waste, ground granulated blast-furnace slag (GGBFS), and red mud (RM) can also be used to further decrease the materials’ carbon footprint [6].

The interest in using highly porous geopolymers (geopolymer foams-GFs) in high-added value applications has grown in recent years, as highlighted in [7]. GFs can be produced by different techniques, including mechanical [8,9] or chemical foaming [10,11], the use of sacrificial fillers [12], emulsion templating [13], or using additive manufacturing [14,15]. Nevertheless, chemical foaming is the most common route. Chemical foaming takes advantage of a chemical reaction occurring between the foaming agent (e.g., H_2_O_2_, aluminum, and silicon) in the alkaline environment. The chemical reaction produces gas bubbles that promote the expansion of the slurry while generating pores, developing a porous matrix structure [16]. When using H_2_O_2_ as the foaming agent, the O_2_ gas released during the decomposition of hydrogen peroxide (as shown in Equation (1)) promotes the foaming of the geopolymer slurry [17]. On the other hand, the reactions of both aluminum (Equation (2)) [18,19] and silicon [19] (Equation (3)) in an alkaline environment result in the release of hydrogen gas, creating pores inside the geopolymer matrix:

(1) $$2H_{2}O_{2}\left(l\right)\to 2H_{2}O\left(l\right)+O_{2}\;\left(g\right)$$

(2) $$2\mathrm{Al}\left(s\right)+2\mathrm{MOH}\left(\mathrm{aq}.\right)+2H_{2}O\left(l\right)\to 2{\mathrm{MAlO}}_{2}\left(\mathrm{aq}.\right)+3H_{2}\left(g\right)$$

(3) $$\mathrm{Si}\left(s\right)+2\mathrm{MOH}\left(\mathrm{aq}.\right)+H_{2}O\left(l\right)\to M_{2}{\mathrm{SiO}}_{3}\left(\mathrm{aq}.\right)+2H_{2}\;(g)$$

During the foaming process, the gas release must be controlled to produce foams with homogeneous pore size distribution. Surfactants are usually required to prevent the coalescence of gas bubbles. They are usually amphiphilic compounds [20] classified as non-ionic, anionic, cationic, and amphoteric [21,22]. The ionic surfactants are considered to provide more enhanced foam stability yet, in the case of geopolymer slurries, which might contain several ions (such as K^+^, Na^+^, Fe^3+^), the non-ionic surfactants might have a better impact since a part of its groups are not electrically charged [23]. The use of cationic surfactants can interact with the negatively charged framework of geopolymers. In contrast, anionic surfactants can interact with the positively charged species in the geopolymer slurries, which might affect the foamability of the foams and, as a result, the properties of the produced bodies. Different surfactants have been employed in the production of porous geopolymers, including sodium dodecyl sulfate (SDS) [24,25,26], sodium lauryl sulfonate (SLS) [27], Tween 80 [28,29], Hostapur OSB [30], and Triton X100 [26,29]. Nevertheless, in most cases, the surfactant dosage was kept constant [26,30,31]. For example, Perumal et al. [31] studied the role of the surfactant (cetyl trimethyl ammonium bromide, cationic or Triton X-100, non-ionic) coupled with H_2_O_2_ (blowing agent) while maintaining the surfactant concentration constant at 0.5 wt.% (of the binder). Results showed similar water absorption values between the reference (composition prepared with a foaming agent but without surfactant) and the samples prepared with surfactants. However, the foams’ pore size had been reduced by the addition of surfactants, and the mechanical strength increased due to the bubble stabilization promoted by the surfactant that prevents bubble coalescence. In another study [26], the influence of the surfactant nature in the production of metakaolin-based GFs has been studied, though again this has been done using a constant surfactant dosage. The surfactants (anionic, cationic, and non-ionic) affected the viscosity of the GFs and the morphology of the samples produced. Based on the study, the order of different viscosities from highest to smallest is sodium dodecyl sulfate (SDS, anionic), CTAB (cationic), Triton X-405 (non-ionic), Triton X-100 (non-ionic), and Triton X-114 (non-ionic). However, according to the authors, the type of surfactant had no pronounced effect on the compressive strength values, despite the different porosity of the foams. Nonetheless, the samples prepared with Triton X-100 showed slightly higher strength values. The authors then attempted to optimize the concentration of this surfactant (ranging from 0.5 to 1.5 × 10^−6^ mol/g of paste) by measuring the compressive strength of specimens. The compressive strength did not show a specific trend when increasing the surfactant dosage. The compressive strength decreased as the Triton X-100 jumped from 0.5 to 1.0 × 10^−6^ mol/g while increasing as the surfactant increased to 1.5 × 10^−6^ mol/g. This was associated with the increased viscosity that can prevent the foaming process. Another recent work evaluated the effect of the surfactant agent on the microstructure of porous metakaolin geopolymers [24]. This study demonstrated that the type of surfactant significantly influences the pore microstructure; though again, all the compositions were prepared at a fixed amount of surfactant, and as a result, the influence of this parameter remains mostly unexplored. The impact of the surfactant amount (Sika^®^ Lightcrete powder), ranging from 1.0 wt.% to 5.0 wt.%, has been studied in [9]. However, it should be noted that the samples prepared without a foaming agent increased with the surfactant inclusion compared with the reference (0 wt.% surfactant), reaching a maximum at a 2 wt.% incorporation. On the contrary, the compressive strength decreased substantially from 21 MPa (reference) to 3.6 MPa (composition containing 5 wt.% surfactants). Recently, the pore structure of metakaolin/fly ash geopolymers prepared using sodium oleate (anionic) as a surfactant and H_2_O_2_ as a foaming agent was studied [32]. The effect of the surfactant concentration on the expansion characteristics of the slurry and the pore structure of the specimens was investigated. Results showed that the surfactant content modifies the bubble size and stability in the H_2_O_2_ foaming system. However, the pore size did not decrease uniformly with increasing stabilizing agent content. Nevertheless, the number of pores and their connectivity increases as the stabilizing agent content in the compositions rises. These are very interesting findings. However, the impact of the surfactant dosage on the foams’ mechanical and thermal properties has not been considered and will be studied in the present study.

Despite previous investigations, the role of the content and type of surfactant on the pore size distribution and the nature of the produced pores (open or closed) is, to date, not fully perceived, as highlighted in recent reviews on the topic [7,20]. The present study intends to fill the research gap by performing a detailed investigation concerning the impact of the dosage and type of surfactant on the properties of red mud-containing geopolymer foams. The foams were prepared using aluminum powder as a foaming agent and a pre-determined amount of surfactant. Three surfactants (i.e., Hostapur OSB (anionic), Sodium dodecyl sulfate (SDS, anionic), and Triton^®^ X-114 (non-ionic) were studied. The effect of the surfactant (dosage and type) and the amount of foaming agent (Al) on the geometric density, water absorption (through immersion and capillary tests), compressive strength, thermal conductivity, and the microstructure of the produced foams were studied.

## 2. Materials and Methods

### 2.1. Materials

In this study, red mud (RM) supplied by a bauxite mining company and metakaolin (MK) (Univar^®^, Argical^TM^ M1200S, Seattle, WA, USA) were used as raw materials. Sodium silicate (SS) (silica modulus = 3.1, H_2_O = 62.1 wt.%, Quimialmel, Albergaria-a-Velha, Portugal) and a sodium hydroxide solution (SH) (ACS reagent, 97%; Sigma Aldrich, Saint Louis, MO, USA) were used as alkali activators. A 12 M solution was prepared by dissolving sodium hydroxide pellets in deionized water. Al powder (95% purity, product code 7429-90-5, Roth, Germany) with a particle size of 50 µm was used as a chemical foaming agent. Hostapur OSB, SDS, and Triton^®^ X-114, whose properties are given in Table 1, were used as surfactants.

### 2.2. Preparation of the Specimens

Geopolymer pastes were synthesized according to the targeted compositional ratios of RM:MK = 2:3, SH:SS = 1:3 (26.6 wt.% MK, 17.8 wt.% RM, 41.7 wt.% SS and 13.9 wt.% SH). It should be noted that the binder composition was intentionally kept constant to allow a direct comparison between the synthesized compositions. The mixture design was defined by reviewing preliminary tests in which the ratio between the RM and MK (from 40 to 50 wt.%) and the SS to SH ratio (from 4:1 to 3:1) was varied. The various samples were characterized by their compressive strength. These tests showed that using 40 wt.% red mud (in the solid fraction) coupled with a sodium silicate:sodium hydroxide 3:1 led to the highest mechanical strength. Therefore, this composition was selected for this study.

To synthesize the geopolymer foams (GF), firstly, SH and SS were mixed for 5 min. Secondly, the solid precursors and the activating solution were mixed for 10 min. Lastly, Al powder (at 0.05, 0.075, and 0.10 wt.% of the binder) and the surfactant (at 0.025, 0.05, and 0.075 wt.% of the binder) were added and mixed for 2 min. Control specimens were also produced without a foaming agent or surfactant to measure the true density of the geopolymers (helium pycnometer). After blending, specimens were poured into prismatic molds (20 × 20 × 40 mm^3^ for compressive strength and water absorption tests and 40 × 40 × 40 mm^3^ for thermal conductivity), sealed, and cured in a climate chamber at 40 °C and 65% relative humidity for 24 h. Thereafter, specimens were removed from the mold and left under ambient conditions until the relevant test day.

### 2.3. Characterization Methods

Particle size distribution of MK and RM was conducted by laser scattering (Coulter Corporation LS230 analyzer, Miami, FL, USA). To determine the chemical composition of raw materials, X-ray fluorescence (XRF- Philips X’Pert PRO MPD spectrometer, Malvern Panalytical, Malvern, UK) was used. The mineralogical compositions of GFs after 28 curing days were also evaluated using X-ray diffraction analysis (XRD, Panalytical, Almelo, Netherlands), and phase identification were executed using PANalytical X’Pert HighScore Plus PRO^3^ software (Malvern Panalytical, Malvern, UK). Fourier-transform infrared spectroscopy with attenuated total reflection (FTIR, Tensor 27 spectrometer, Bruker, Middlesex, MA, USA), resolution: 4 cm^−1^, wavenumber range: 4000–350 cm^−1^) was also used to characterize the solid precursors, and the reference geopolymer (composition prepared without foaming agent and surfactant). Moreover, scanning electron microscopy (SEM—S4100 equipped with energy dispersion spectroscopy, Hitachi, Tokyo, Japan; EDS—model Quantax 400, Bruker, Billerica, MA, USA) was utilized to characterize the microstructure of the foams. After the SEM images were acquired, ImageJ software (Version 1.53a, National Institutes of Health, Montgomery, MD, USA) was used to measure the pore size distribution of selected foams (prepared with 0.05 wt.% Al and a varying amount of the three studied surfactants). The specimens’ morphology was also examined using an optical microscope (Nikon, H550S, Tokyo, Japan). 

The water absorption test was carried out to measure the percent increase in mass of the prismatic samples after immersing the specimens in water for 24 h at ambient temperature. In addition, the capillary water absorption test was carried out according to standard EN 1015-18 by using prismatic samples (20 × 20 × 40 mm^3^).

After the samples were dried in an oven at 60 °C for 24 h, their geometric density [$\rho_{g}$ (kg/m^3^)] was calculated by the ratio of dry mass to volume. Three prismatic samples from each series were used. The Archimedes method and He-gas pycnometry (Ultrapyc 5000 Micro, Anton Paar) were used to determine the specimens’ open and total porosities, respectively. Using a He-gas pycnometer, true density [$\rho_{t}$ (kg/m^3^)] was determined, and total porosity (TP) values were calculated using Equation (1). While using the Archimedes method, the samples were first dried in an oven at 60 °C for 24 h. The dry mass (*m_d_*) of the specimens was measured, and then they were immersed in distilled water. After 24 h, the saturated mass (*m_s_*) of the samples was measured. After water impregnation, the mass of the specimens while suspended in water (*m_w_*) was determined. The open porosity (OP) values were calculated using Equation (2):

(4) $$TP\left(\%\right)=\frac{\rho_{t}-\rho_{g}}{\rho_{t}}\times 100\;$$

(5) $$OP\left(\%\right)=\frac{m_{s}-m_{d}}{m_{s}-m_{w}}\times 100$$

The compressive strength of the foams, measured 28 days after synthesis, was performed using a Universal Testing Machine (Shimadzu AG-25 TA, Kyoto, Japan) at a 0.5 mm/min load rate. The given values are the averages of three different prismatic samples’ measurements. Furthermore, the thermal conductivity of GFs was measured using a heat flow meter apparatus according to ASTM C518-04 standard, using three samples (40 × 40 × 40 mm^3^) per mix. The sample is placed amid two parallel plates, which are adjusted to 55 °C and 40 °C between the upper and bottom plates, respectively. The thermal conductivity values of the samples were found by applying a unidirectional heat flux throughout the sample.

## 3. Results and Discussion

### 3.1. Characterization of Raw Materials

**Figure 1:**
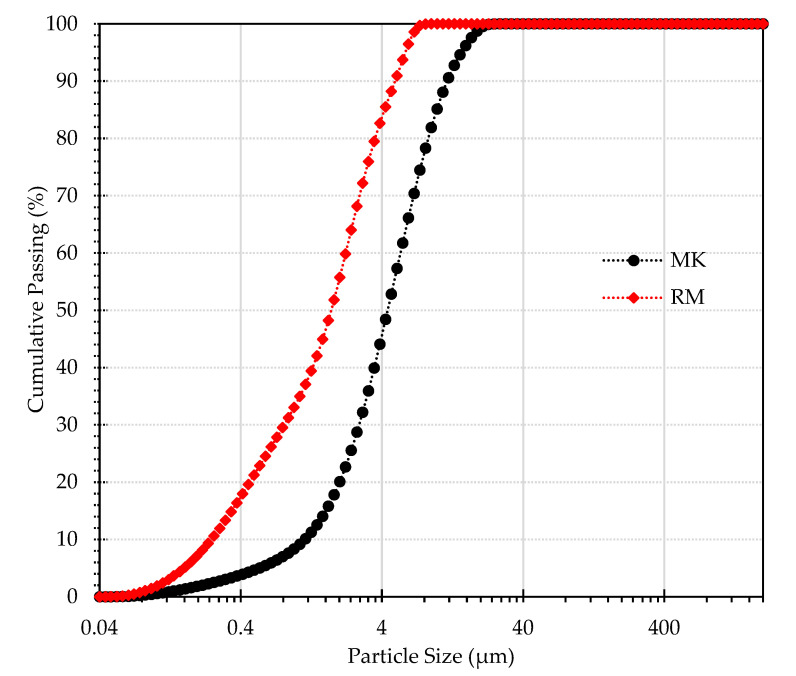
The particle size distribution (PSD) of raw materials.

The chemical composition of the solid precursors is shown in Table 2. RM was collected as a slurry containing a high-water content. Therefore prior to its use in the production of the geopolymers, the RM was dried at 100 °C (24 h), milled (aiming to deagglomerate the RM particles), and sieved to attain a particle size below 75 µm. As for MK, only the drying step was performed. As a result, MK has coarser particles compared with the RM, the median particle size (D50) being 4.4 μm and 1.7 μm, respectively, for MK and RM. The differences between the particle size distribution of the used precursors can be observed in Figure 1. Despite the differences in the particle size distribution of the precursors, which might affect their reactivity in the alkaline medium, no attempt was made to control their size as the mixture design (RM:MK) was kept constant throughout the experiments.

The chemical composition of RM shows that this waste is mainly composed of iron oxide (approximately 48%), also containing significant amounts of Al_2_O_3_, TiO_2_, SiO_2_, and Na_2_O. MK is a rather pure aluminosilicate resource, the silica, and alumina accounting for roughly 92 wt.% of the precursor chemical composition. 

**Figure 2:**
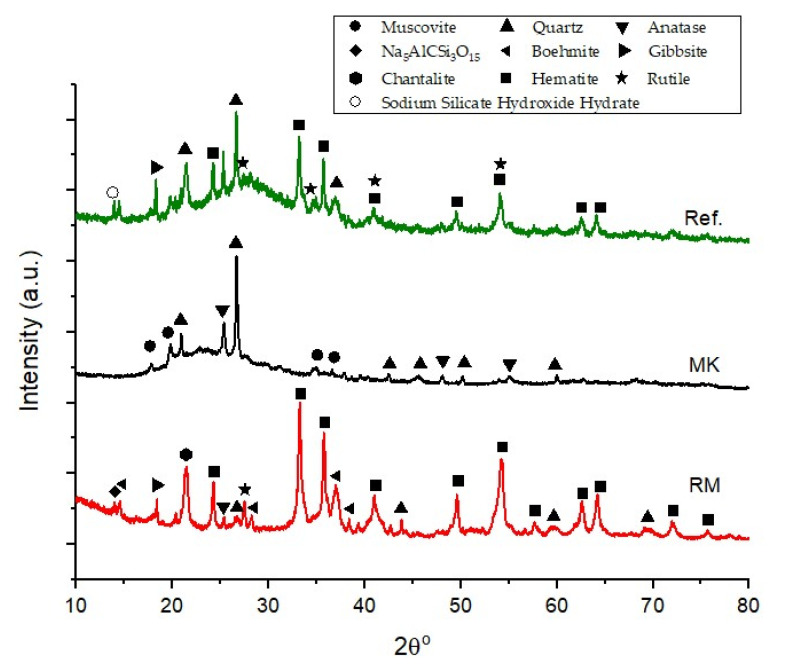
XRD patterns of the raw materials and red mud geopolymer (reference) prepared without a foaming agent or surfactant.

The XRD patterns of the raw materials, presented in Figure 2, suggest that MK has a higher amorphous content when compared to RM, as demonstrated by the characteristic protuberance from 15° to 30° 2θ. Nonetheless, the XRD diffractogram of MK also shows the presence of crystalline phases, such as quartz (SiO_2_, PDF 00-046-1045), muscovite (K_0.77_Al_1.93_(Al_0.5_Si_3.5_O_10_)(OH)_2_, PDF 01-070-1869), and anatase (TiO_2_, PDF 01-084-1285). RM, on the other hand, is a highly crystalline precursor, the main observed phases being hematite (Fe_2_O_3_, PDF 00-033-0664) and aluminum hydroxides, including boehmite (AlO(OH), PDF 00-021-1307) and gibbsite (Al(OH)_3_, PDF 04-013-6979). Na_5_AlCSi_3_O_15_ (Sodalite, PDF 00-015-0469) and rutile (TiO_2_, PDF 00-021-1276) were also detected in RM pattern. In the pattern of reference geopolymer composition (coded as Ref.), a new amorphous phase was observed between 20° and 35° 2θ. The appearance of a new amorphous phase confirms that the geopolymerization reaction occurred during the material’s synthesis [24].

**Figure 3:**
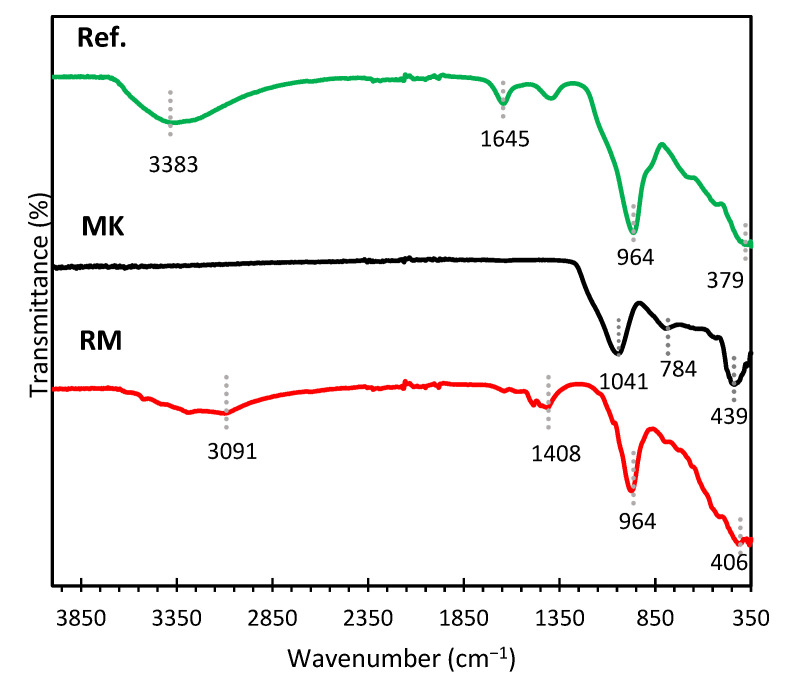
FTIR spectra of raw materials and reference sample.

The FTIR spectra of the reference geopolymer, MK, and RM are shown in Figure 3. It can be noticed that the FTIR pattern of MK had three broad features at approximately 1041 cm^−1^, 784 cm^−1^, and 439 cm^−1^. The densest, asymmetrical, broad feature at 1041 cm^−1^ indicates the stretching of Si-O bonds in the amorphous MK structure [33,34,35]. The vibrations of the AlO_4_ tetrahedra in MK were attributed to the broad band located at about 784 cm^−1^ [33]. The Al-O-Si bridge of aluminosilicates was assumed to be connected to the band at 439 cm^−1^ [33,36]. A prominent band was visible in the RM spectrum at 3091 cm^−1^ in the hydroxyl-stretching (O-H) area [34]. This was most likely caused by the water content of the RM [37]. The H-O-H stretching vibration in RM was related to the band at 1408 cm^−1^ [34,35]. For the RM spectrum, the characteristic band at 964 cm^−1^ corresponding to the asymmetric stretching vibrations of Si-O-Si and Si-O-Al was observed [35]. Al-O and Fe-O bond groups were contributed by the peak of RM at roughly 406 cm^−1^ [38]. For the reference geopolymer, the broadband at 3383 cm^−1^ detected in the spectrum can be assigned to the stretching vibration of structural and free water [24]. Likewise, the small band of 1645 cm^−1^ is attributed to the bending vibration of free water [39]. The main band of the reference geopolymer is located at 964 cm^−1,^ and this band is an important indicator of the matrix describing the asymmetric stretching vibrations of Si-O-Si or Si-O-Al bonds because of TO_4_ (T: Si or Al) reorganization during the geopolymerization [36,40].

### 3.2. Microstructural Characterization of GFs

The size, morphology, and volume of the pores are known to affect the properties of GFs. Round and homogeneous pores improve the thermal insulation properties [41,42], while pores showing irregular shapes coupled with a wide size distribution create interconnected air channels that might improve the acoustic properties of these materials endowing their use in sound insulation applications [41,42,43]. Therefore, the precise control of the morphology of the pores and their connectivity is of the utmost importance to extend the application range of GFs.

**Figure 4:**
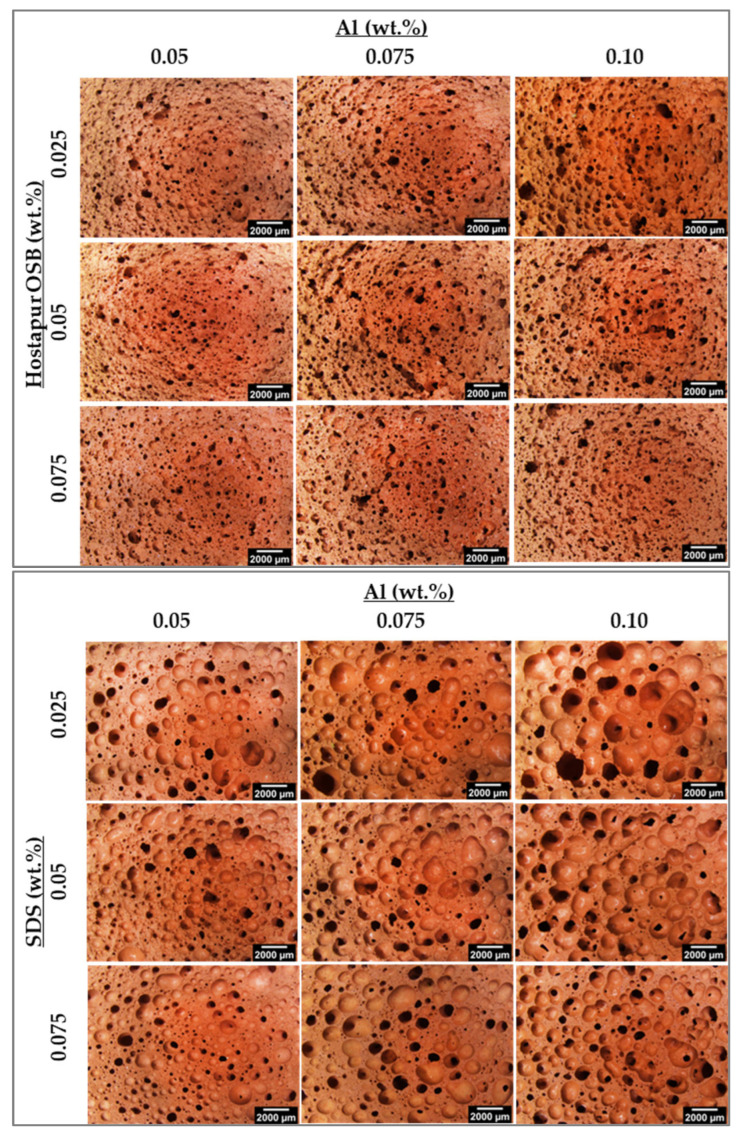

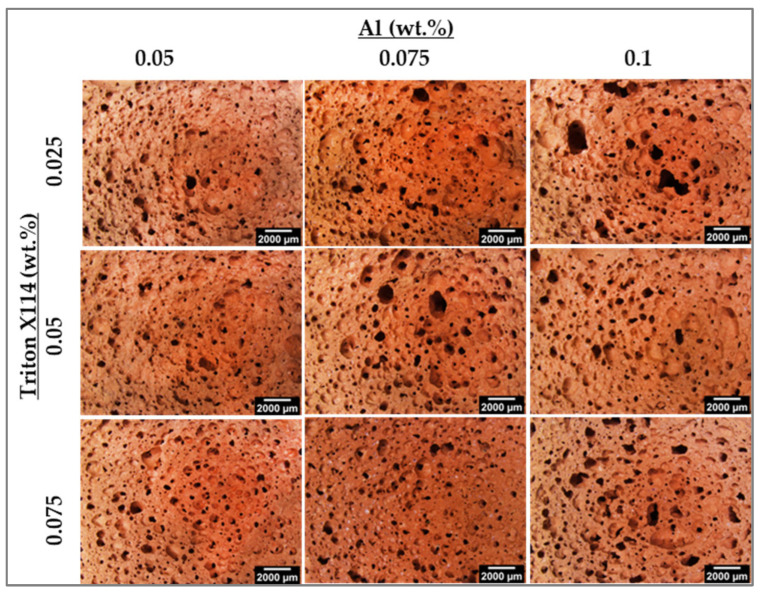
Optical microscope photographs of samples produced using various surfactants.

**Figure 5:**
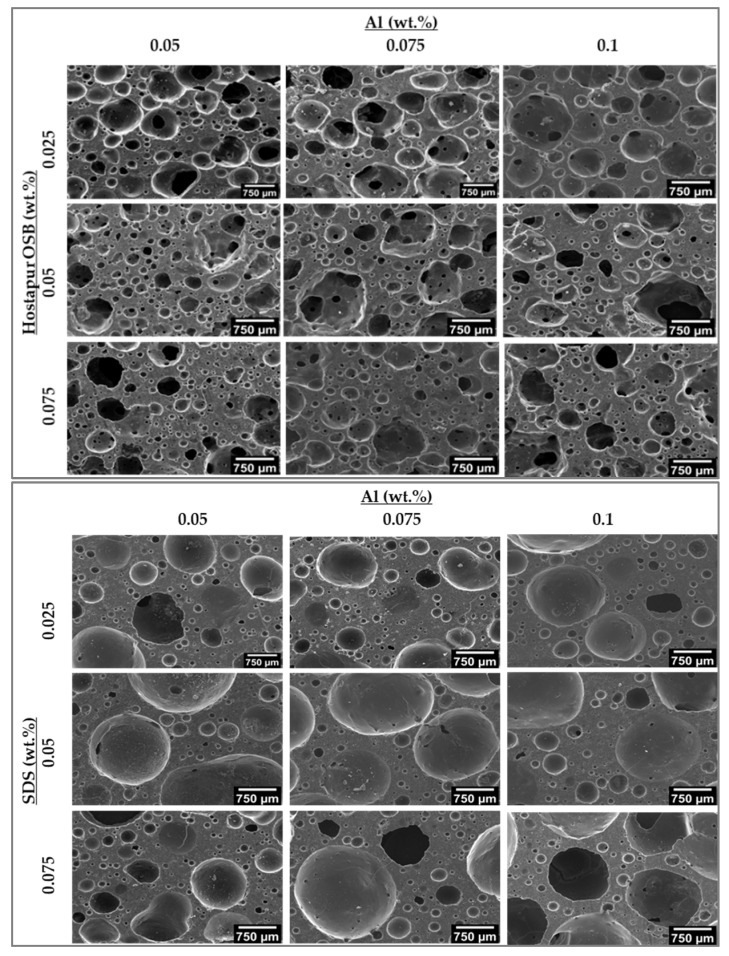

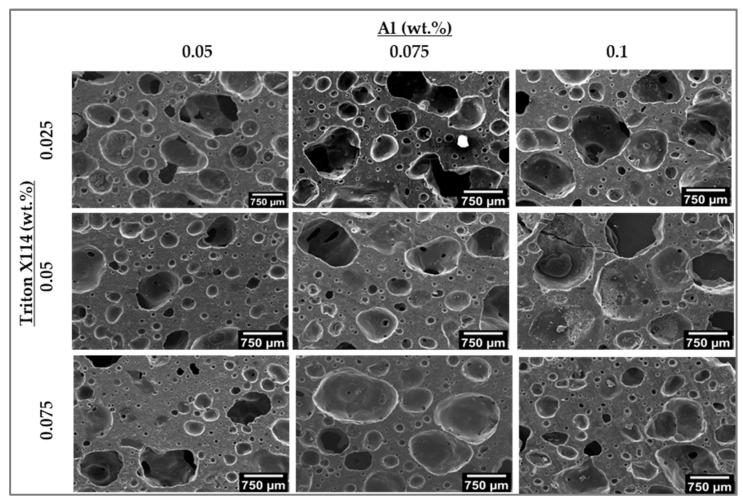
SEM micrographs of samples produced using various surfactants.

Optical and SEM micrographs of the GFs prepared in the present study and produced with three different surfactants and various amounts of Al powder (0.05–0.1 wt.%), used here as the foaming agent, are presented in Figure 4 and Figure 5, respectively. Different features can be depicted from the micrographs depending on the nature and concentration of the surfactant in the compositions. Figure 4 shows that in the case of Hostapur OSB, higher amounts of the surfactant increase the number of produced pores but simultaneously decrease their average size, this being particularly noticeable at the lower Al incorporation level (0.05 wt.%). As the amount of Al increases, the connection between the pores also increases (see discussion in Section 3.2), and the pore shape becomes irregular. During the reaction of Al in an alkaline environment, as the H_2_ gas released in the geopolymer matrix increased, the pores might be enlarged and merged with other pores. When pores get excessively large, they collapse and connect, causing pore coalescence [9]. The samples prepared with SDS show a rather different pore morphology. In this set of samples, much bigger pores are visible compared to those prepared using Hostapur OSB. Similar to what was reported for the Hostapur OSB, at the lower Al incorporation (0.05 wt.%), increasing the amount of SDS decreases the size of the produced pores, suggesting that the use of higher amounts of the surfactant can, at least partially, dilute the coalescence of the gas bubbles. Nonetheless, this feature is less relevant in the compositions prepared with higher amounts of the foaming agent. The micrographs also show differences in the shape and connectivity of the pores in samples prepared with SDS compared with those produced with Hostapur OSB, the former samples exhibited round-shaped [25], but mostly closed pores, while in the latter, irregular and open pores were observed. Small and closed pores may contribute to thermal insulation [44], while open pores will benefit wastewater treatment applications [35]. The rheology and setting time of the mixture can influence stabilizing of the pores. If the geopolymer slurry has a viscous and rapid hardening structure, pores can be retained in the matrix without connecting and becoming coarser [45]. As for the samples produced with Triton X114, the impact of the surfactant dosage on the pore size and connectivity does not show a clear tendency, being dependent on the Al content. 

**Figure 6:**
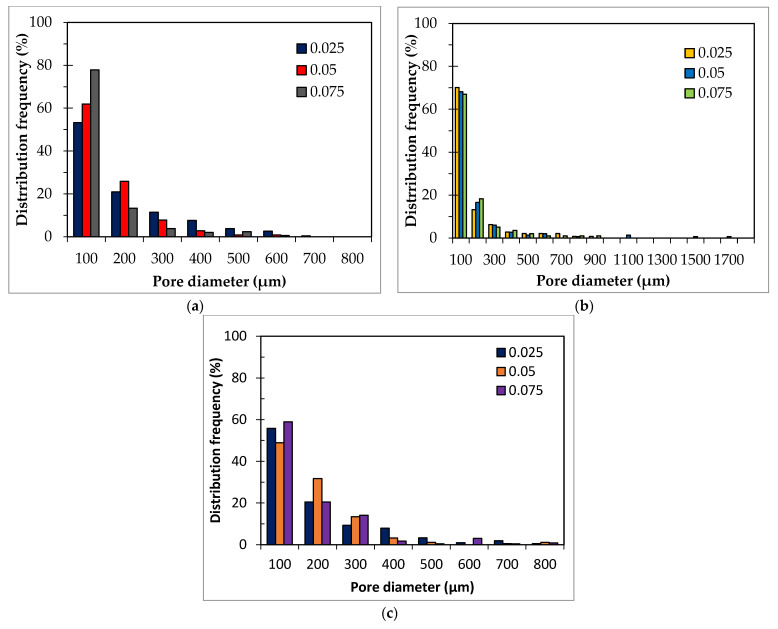
Pore size distribution (measured by image analysis of the SEM images shown in Figure 5) of GFs prepared with 0.05 wt.% Al, and 0.025, 0.05 and 0.075 wt.% surfactants: (**a**) Hostapur OSB, (**b**) SDS, and (**c**) Triton X114.

To further characterize the pore size distribution of the various foams image analysis of selected SEM images, corresponding to the samples produced using the lowest Al content (0.05 wt.%) and the three studied surfactant concentrations (0.025, 0.05, and 0.075 wt.%), shown in the first column of Figure 5, was performed. Figure 6a shows that when using Hostapur OSB as a surfactant, the pore size decreases as the surfactant content increases from 0.025 to 0.075 wt.%. In line with the previous remarks regarding the impact of the amount of this surfactant, the pore size distribution curves show a much higher frequency of small-sized pores (below 100 µm) in the highest containing Hostapur OSB composition (78%) compared with the lowest Hostapur OSB counterpart (53%). Figure 6b shows the presence of much bigger pores in the SDS compositions compared to those seen in the Hostapur OSB. Here, the impact of the amount of the surfactant is less pronounced, with a minor increase in the frequency of small-sized pores (below 200 µm) from 83% to 85% as the surfactant content increases from 0.025 to 0.075 wt.%. Figure 6c shows that the frequency of pores below 100 µm in the composition containing 0.025 wt.% Triton X114 is 56%, decreasing to 49% when the surfactant content rises to 0.05 wt.%, then increasing to 59% as the surfactant increases to 0.075 wt.%.

These results show that Hostapur OSB leads to the production of a narrower pore size distribution in comparison with the other studied surfactants. One possible explanation might be the distinct viscosity of the slurries. In fact, previous studies have shown that the viscosity of geopolymeric slurries is strongly affected by the surfactant type [46]. This topic will be studied in a follow-up investigation.

### 3.3. Physical and Mechanical Characterization

Table 3 presents the geometric density, total, open and closed porosity, water absorption, and thermal conductivity of the various foams. In the previous section, we saw that the content of Hostapur OSB affects the pore size and their distribution (see Figure 4, Figure 5 and Figure 6) in the foams, with a higher number of small-size pores being observed when rising the dosage of this surfactant. Nevertheless, the impact of the surfactant content on the properties of the produced foams seems to be less pronounced than the impact of the foaming agent (Al). For example, the water absorption of the foams prepared with 0.025 wt.% surfactant increases from 64.0% (0.05 wt.% Al) to 80.4% when using 0.075 wt.% Al. On the contrary, when setting the Al content to 0.05 wt.% and varying the surfactant content, the water absorption of the samples is mostly unaffected. Surfactants stabilize the liquid-gas interface and prevent or at least mitigate pore coalescence [31,46]. Previous literature suggested that while the use of a surfactant can reduce the size of the produced pores in comparison with the non-containing surfactant composition, the water absorption of the samples is very similar [31]. However, contradicting observations have been reported in [9]. In this study, an increase in the water absorption of the surfactant-containing compositions was observed compared with the reference. Our results (Table 3) show that, at least under the studied compositional range, the impact of the surfactant nature and concentration in the open porosity of the samples, and thus in the water absorption, is minor compared to the effect of the foaming agent content. The exception to this general trend is the significant drop in the water absorption for the samples prepared with 0.075 wt.% of Al and 0.05 wt.% surfactant (72.9%) compared to their lower containing surfactant counterparts (80.4%, 0.025 wt.% Hostapur OSB). Nonetheless, the higher value seen in lower-containing surfactant composition is abnormal and should be considered with caution. In fact, the open porosity of these specimens is similar to that seen in the higher-containing surfactant compositions. 

Table 4 contains data collected from various literature studies focusing on producing MK or RM-containing GFs. In the current study, the water absorption of the foams varied between 42% and 80%. The lower and upper limits are higher than the values reported for other RM/MK foams (26–41%) [47] and MK-containing foams (30–60% [48]; 34–50% [49]). Nevertheless, higher water absorption values were seen in (42–112%) [50] and (39–92%) [51]. 

**Figure 7:**
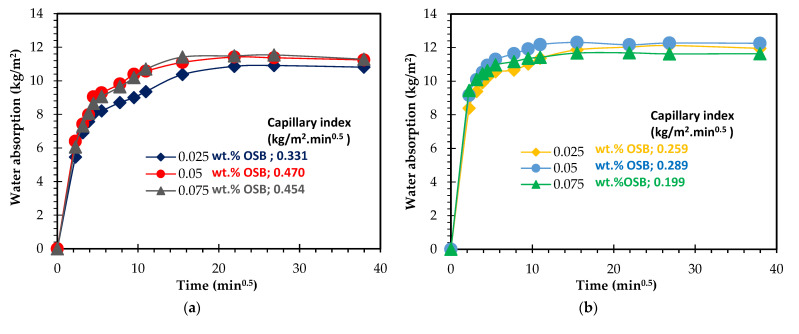

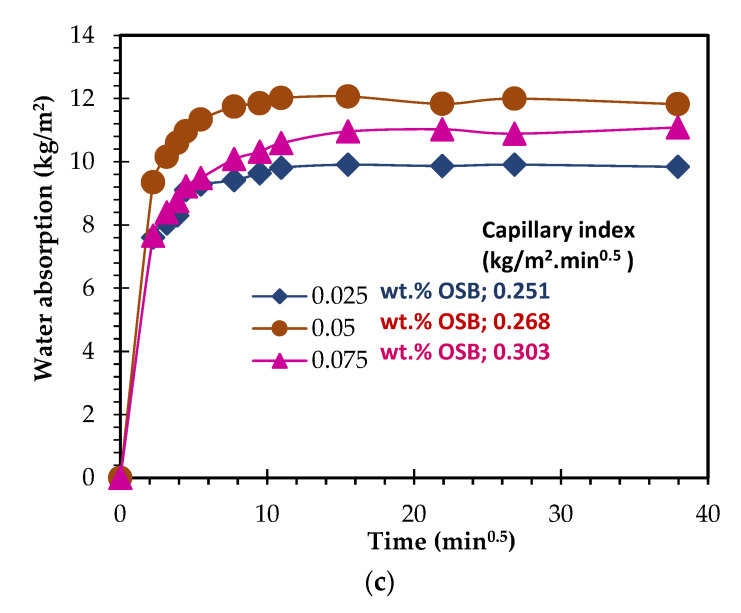
Capillary water absorption and index of GFs prepared with different content (0.025, 0.05, and 0.075 wt.%) of Hostapur OSB and (**a**) 0.05 wt.% Al, (**b**) 0.075 wt.% Al, and (**c**) 0.1 wt.% Al.

To provide additional insights regarding the pore size distribution of the specimens, capillarity water absorption tests were carried out, and the results are presented in Figure 7. The samples were prepared with 0.05 wt.% Al (Figure 7a) shows a rather fast and similar water uptake within the first 10 min, independent of the surfactant content. Thereafter, gentler water absorption by the samples is observed, the samples reaching a plateau after 4 h. Despite the similarities between the curves, Figure 7a shows that the sample prepared with the lowest amount of surfactant (0.025 wt.%) has a lower water capillary index (0.33 kg/m^2^.min^1/2^) compared to the composition prepared with a higher dosage (e.g., 0.45 kg/m^2^.min^1/2^–0.075 wt.% Hostapur OSB), which suggests the presence of a higher number of capillary pores in the latter. This finding is in line with the optical micrographs presented in Figure 4 and the pore size distribution measured by image analysis (see Figure 6), which show a decrease in the size of the produced pores as the surfactant content rises. Figure 7b shows that as the foaming agent content rises to 0.075 wt.%, faster and higher water uptake by the porous samples is observed. In these samples, the impact of the surfactant dosage is minor, and this conflicts with the differences seen in the water absorption reported in Table 3 (discussed above). This feature is not fully perceived and will be studied in future work. Figure 7c, corresponding to the samples prepared with the highest studied Al amount, reveals a significant difference between these compositions not only in the water absorption rate but also in the water absorption level (measured after 24 h). The composition containing 0.05 wt.% Hostapur OSB shows a much faster water absorption than the other samples. In fact, this is seen immediately within the first 5 min. These specimens also present higher water absorption by capillarity (11.8 kg/m^2^) compared to the lower surfactant-containing composition (9.8 kg/m^2^–0.025 wt.% OSB), this being in line with the slightly higher water absorption measured by immersion (77.4% vs. 74.8%, reported in Table 3) and with the higher open porosity (47.7% vs. 44.0%).

Table 3 shows that the use of SDS as a surfactant leads to the production of specimens with much lower open porosity compared to the Hostapur OSB, regardless of the Al content. The open porosity of the SDS-containing specimens ranges from 28.3 to 41.2% while being comprehended between 39.3 and 47.7% in those prepared with Hostapur OSB. This is an interesting finding showing that the nature of the surfactant can be an interesting way to tailor the open/closed porosity of GFs. According to the literature, the pore structure can be affected by the viscosity of the slurry, the geopolymerization rate, and the properties of the surfactants [52]. The XRD patterns of the foams produced with the distinct surfactants (not included for the sake of brevity) were similar, suggesting that the different surfactants did not modify the geopolymerization degree. Therefore, the differences between the Hostapur and the SDS systems are probably due to the different viscosity of the pastes. Indeed, the viscosity of SDS-containing MK geopolymer slurries has been reported to be much higher than those seen with Triton X114 [26], while the use of Hostapur can improve the workability of mortars [53]. A follow-up study to evaluate the impact of the surfactant nature on the fresh-state properties (e.g., yield stress, viscosity, setting time, expansion rate) is suggested to further clarify the role of the nature of the surfactant in the pore size distribution of the metakaolin/red mud foams.

**Figure 8:**
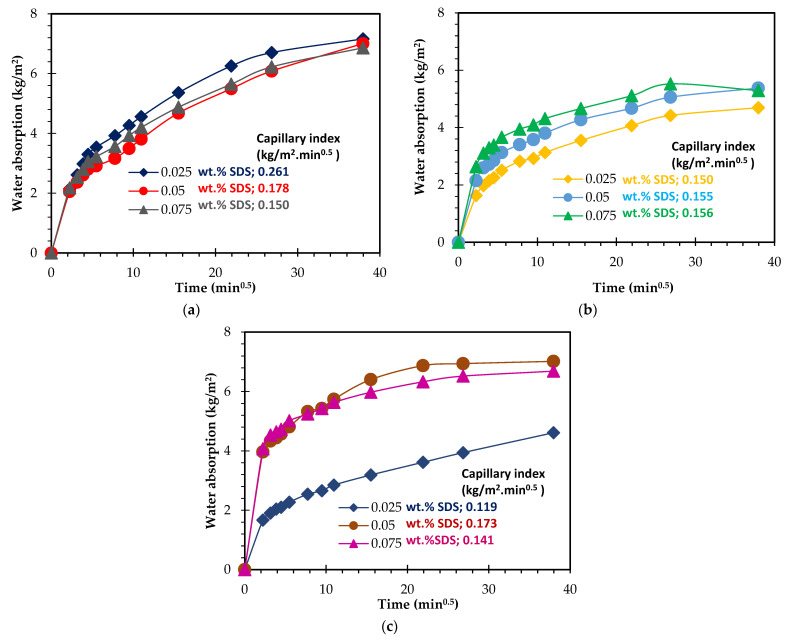
Capillary water absorption and index of GFs prepared with different content (0.025, 0.05, and 0.075 wt.%) of SDS and (**a**) 0.05 wt.% Al, (**b**) 0.075 wt.% Al, and (**c**) 0.1 wt.% Al.

**Figure 9:**
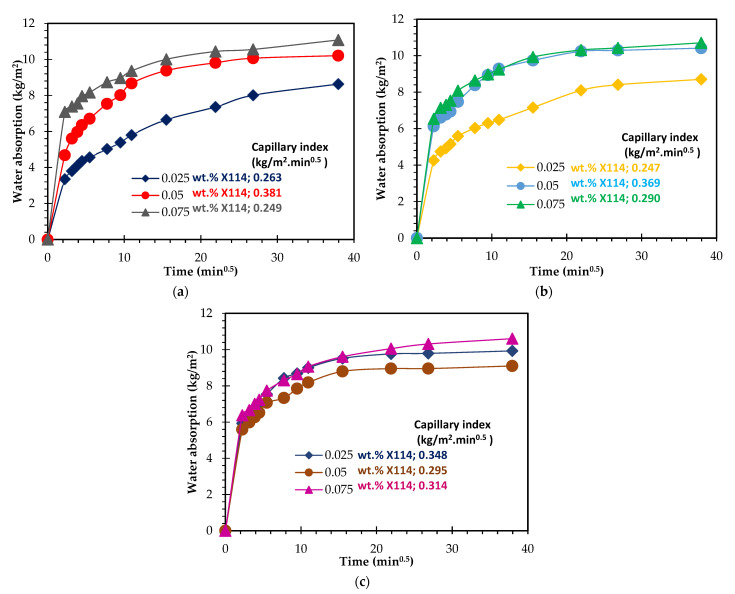
Capillary water absorption and index of GFs prepared with different content (0.025, 0.05, and 0.075 wt.%) of Triton X114 and (**a**) 0.05 wt.% Al, (**b**) 0.075 wt.% Al, and (**c**) 0.1 wt.% Al.

Similar to what was observed with the Hostapur OSB, the impact of the SDS content is minor when compared with the Al content when the Al concentration is below 0.075 wt.%. However, this is not the case for the higher containing Al content foams, as shown by the much higher water absorption and open porosity values. The composition containing 0.050 wt.% SDS has water absorption of 72.1% and open porosity of 41.2%, respectively, being 14.1% and 8.9% higher than when using 0.025 wt.% SDS. These findings are supported by the water capillarity tests shown in Figure 8. Minor differences are seen between the compositions containing various SDS contents but prepared with a fixed amount of Al when the Al contents are ≤0.075 wt.% (Figure 8a,b). Nonetheless, a major impact on the water absorption rate and the amount is visible for the three studied compositions when 0.10 wt.% Al is employed in the foams synthesis (see Figure 8c), which clearly shows that the effect of the surfactant dosage is highly dependent on the amount of the foaming agent. As for Triton X114, the data shown in Table 3 does not reveal a specific trend regarding the impact of the surfactant concentration on the studied properties. As mentioned above regarding the SDS, the effect of the Triton X114 dosage seems to be dependent on the foaming agent amount. Nevertheless, the capillarity water absorption curves, shown in Figure 9, provide some insights into the specimens’ pore size distribution and connectivity between the pores. Figure 9a,b suggests that using a higher amount of this surfactant generates a higher number of capillary pores, thus explaining the steeper slope observed in the curves. Nevertheless, this feature is not observed in the highest Al-containing foams (Figure 9c). Another noteworthy finding is that the open porosity range observed when using this surfactant (36.5 to 41.3%) is much narrower than those seen for both the SDS (28.3 to 41.2%) and the Hostapur OSB (39.3 to 47.7%). The data presented in Table 3 also shows that the highest total (82.2%) porosity and the lowest density (400 kg/m^3^) values were observed when using 0.025 wt.% SDS coupled with the highest studied amount of Al (0.10 wt.%), while the lowest total (71.6%) porosity and the highest density (630 kg/m^3^) values were observed when combining 0.05 wt.% of Triton X114 with 0.05 wt.% Al. When previous studies are examined (Table 4), it is seen that the combined use of MK and RM is not common. The lowest geometric density (400 kg/m^3^) reported here is lower than those seen in [11,47,49,50,51,54,55], and in addition, the compressive strength of this composition (1.3 MPa) is higher than that reported for [56,57].

The specimens’ thermal conductivity depended on the foaming agent content, with higher amounts generally decreasing the thermal conductivity of the foams. The effect of the surfactant dosage was highly dependent on the Al content. It should also be noted that the proper combination between the surfactant concentration and the Al content allows the production of foams with thermal conductivity below 0.10 W/m.K, regardless of the type of surfactant. The minimum thermal conductivity value (0.09 W/m.K) is also lower than the values observed in previous studies [49,54,58,59,60,61]. This feature is attributed to the fact that thermal conductivity depends not only on the volume of pores but also on the foams’ pore size distribution and the nature of the pores (closed or open) [62]. In addition, according to the literature, it is also stated that GFs with total porosity >50 vol%, bulk density ≤700 g/cm^3^, low thermal conductivity, and good mechanical strength can be beneficial for thermal insulation applications [19]. It should be noted that in the present study, the highest total porosity (82%) value was obtained higher when compared to the previous studies [11,48,49,51,55,57]. The high porosity exhibited by the RM/MK-based geopolymers also suggests their potential use in other high-added-value applications such as adsorbent materials [63,64] or as pH regulators [65,66].

**Table 4:** Comparison of GFs’ results with previous studies.

Precursors/Surfactant/Foaming Agent	Geometric Density (kg/m^3^)	Total Porosity (%)	Thermal Conductivity (W/m.K)	Water Absorption (%)	Compressive Strength (MPa)	Reference
MK + Biomass FA/Calcium stearate/H_2_O_2_	150–300	72–85	0.06–0.08	-	0.7–2.2	[67]
MK + Biomass FA/-/H_2_O_2_	313	86	0.073	-	0.6	[56]
MK + Biomass FA/Hostapur OSB/Al	430–850	74–87	0.08–0.17	42–112	0.6–4.3	[50]
MK + GGBFS/Commercial surfactant/ H_2_O_2_	260–480	-	0.08–0.14	-	0.5–3.3	[68]
MK + Biomass FA/-/H_2_O_2_	440–910	60–81	0.08–0.17	39–92	0.3–5.0	[51]
MK + Wollastonite/Calcium stearate/Na_2_O_2_	350–600	63–73	0.09–0.12	-	0.4–0.7	[57]
**MK + RM/Hostapur OSB- SDS- Triton X114/ Al**	**400–630**	**71–82**	**0.09–0.13**	**42–80**	**0.9–3.3**	**This study**
MK+ Biomass FA/-/H_2_O_2_	560–1200	42–73	0.10–0.43	34–50	1.2–8.9	[49]
MK + Halloysite clay/-/H_2_O_2_	330–850	-	0.11–0.17	-	1.7–5.2	[58]
MK/Sunflower oil-Canola oil-Olive oil/H_2_O_2_	370–740	66–83	0.11–0.17	-	0.3–11.6	[59]
MK + Waste glass/Triton X100/H_2_O_2_	270–480	77–88	0.11–0.15	-	1.2–5.5	[60]
MK/Tween 80/H_2_O_2_	471–1212	36–86	0.11–0.30	-	0.4–6.0	[54]
MK + Aluminum scrap recycling waste + Waste glass + Steel plant waste/-/-	380–470	83–86	0.14–0.15	-	1.1–2.0	[61]
MK + Ground silica powder/Canola oil/H_2_O_2_	310–680	62–83	-	-	0.4–5.7	[69]
MK + FA/Sodium oleate/H_2_O_2_	370–750	66–83	-	-	1.4–3.2	[11]
MK + Zeolite waste+ Calcium hydroxide/-/H_2_O_2_	450–891	56–67	-	-	1.1–7.2	[55]
MK + RM/-/Al	470–870	74–84	-	26–41	0.7–5.8	[47]
MK + FA/Sodium oleate/Al	490–820	63–78	-	-	1.9–4.4	[11]
MK + Biomass FA/-/H_2_O_2_	550–1200	41–75	-	30–60	0.2–9.0	[48]

**Figure 10:**
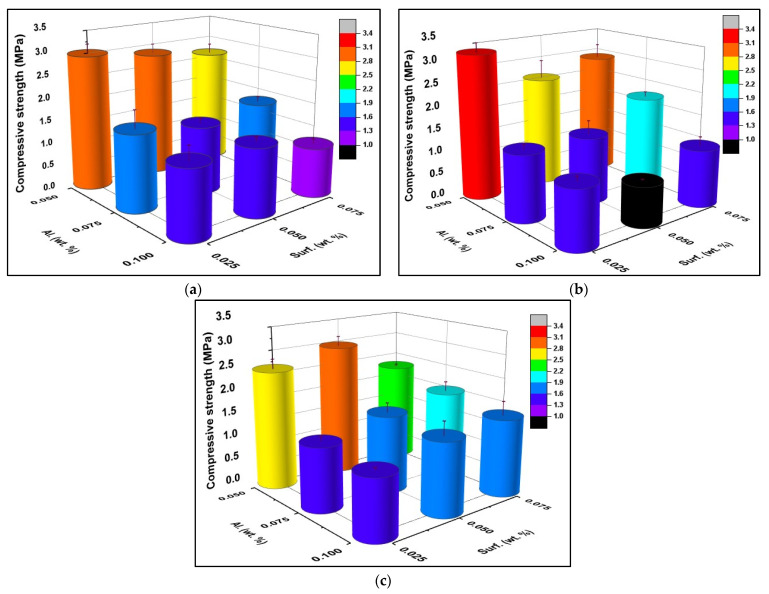
Compressive strength results of samples produced using (**a**) Hostapur OSB, (**b**) SDS, and (**c**) Triton X114 as surfactants.

Figure 10 presents the compressive strength of the various GFs. Not surprisingly, all foams show a decrease in their compressive strength as the foaming agent content in the compositions rises. When the concentration of the foaming agent in the compositions increases, the amount of hydrogen gas released by the reaction of the aluminum powder with water in the alkaline medium rises. As a result, the porosity of the samples increases while their compressive strength decreases. The inverse correlation between the total porosity and compressive strength of porous geopolymer has been demonstrated in the literature [48,49,50].

Moreover, the effect of the surfactant dosage depends on the type of surfactant and the Al content. Higher dosages of Hostapur OSB reduce the specimen’s compressive strength, while no obvious trend is seen for the SDS or the Triton X114. Despite this, the compressive strength of samples prepared with the SDS or the Triton X114 is dependent on the concentration of the surfactant. In fact, the mechanical strength of the specimens can be strongly increased by tailoring the surfactant content. For example, the composition prepared with 0.075 wt.% of SDS and 0.075 wt.% Al shows 40% higher compressive strength (2.1 MPa) compared to the formulation containing 0.025 wt.% surfactant (1.5 MPa). For Hostapur OSB and SDS, the highest compressive strength was reached using 0.025 wt.% surfactant and 0.05 wt.% Al (3.0 MPa—Hostapur OSB; 3.3 MPa—SDS). In the case of the Triton X114, a higher amount of the surfactant was required to reach the maximum strength (2.9 MPa–0.05 wt.% surfactant) as the composition containing 0.025 wt.% surfactants reached only 2.6 MPa (13% lower than the composition prepared with Hostapur OSB and 21% lower than the SDS composition). The maximum compressive strength values obtained for each surfactant type were found to be higher compared to previous studies [56,57,61,67].

The lowest compressive strength values were observed for the compositions containing the highest studied Al amount (0.10 wt.%). Still, the concentration of the surfactant depends on the nature of the stabilizing agent: 0.075 wt.%—Hostapur OSB (1.1 MPa), 0.05 wt.%—SDS (0.9 MPa), 0.025 wt.%—Triton X114 (1.3 MPa), showing that the type and concentration of the surfactant exert a relevant impact on the GFs compressive strength. Notably, the lowest values obtained in this study are higher than the values reported in [56] and [57].

This impact of the nature and content of the surfactant is further illustrated by calculating the foams’ specific strength, the ratio between the compressive strength, and the geometric density (MPa cm^3^/g). The specific strength of the GFs prepared with Hostapur OSB varies between 2.2 and 5.1 MPa cm^3^/g, while in the case of the SDS, higher specific strengths are seen (ranging from 2.2 to 5.6 MPa cm^3^/g). Interestingly, the formulations prepared with Triton X114 show a narrower range of values between 2.5 and 4.5 MPa cm^3^/g. The lowest specific strengths reported here (2.2 MPa cm^3^/g–Hostapur OSB and SDS; 2.5 MPa cm^3^/g–Triton X114) are higher than the lowest values reported for other GFs, including those prepared with 5 wt.% olive oil as a surfactant and 20 wt.% H_2_O_2_ as a foaming agent (0.8 MPa cm^3^/g) [59], 0.05 wt.% Hostapur OSB as a surfactant and 0.08 wt.% Al as a foaming agent (1.4 MPa cm^3^/g) [50], 0.05 wt.% commercial surfactant coupled with 2 wt.% H_2_O_2_ as a foaming agent (1.9 MPa cm^3^/g) [68], which demonstrates the interesting properties of the foams produced in the present investigation.

## 4. Conclusions

To date, most studies dealing with the production of geopolymer foams have neglected the impact of the surfactant type and nature of the pore size distribution, and the physical properties of the produced foams. In the present work, the effects of the surfactant type (Hostapur OSB, SDS, and Triton X114) and dosage (0.025, 0.05, and 0.075 wt.%) on the physical, mechanical, and microstructural properties of GFs prepared using aluminum as a foaming agent were investigated.

The type of surfactant influenced the pore size and morphology of the foams. Results show that using SDS favors the formation of large-sized and spherical pores with low connectivity (e.g., 49.9% closed pores in the sample produced with 0.025wt.% SDS). On the contrary, the specimens produced with Hostapur OSB show smaller-size pores and higher pore connectivity, as demonstrated by the samples’ much higher open porosity (up to 47.7%) and water absorption (up to 80.4%) values. In addition, in the Hostapur OSB samples, the shape of the pores was dependent on the surfactant content, increasing the surfactant amount and distorting the spherical shape of the pores. Besides the distinct porosity, results also show that the specimens’ compressive strength was affected by the nature of the surfactants and their incorporation content, with higher dosages of Hostapur OSB reducing the specimen’s compressive strength (from 3 MPa to 2.6 MPa) at low Al contents (0.05 wt.%). As for the SDS and the Triton X114 containing compositions, no clear tendency is seen, but the mechanical strength of the specimens’ can be increased by up to 40% by tailoring the surfactant content. These results suggest the feasibility of adjusting the open/closed porosity of foams and their mechanical performance by tuning the concentration and nature of the surfactant, which might extend the application range of the foams to thermal and acoustic insulators, and also to wastewater treatment systems in which high open porosity values are required for the adsorbent material. Notably, the foams were produced using significant amounts of hazardous and unexplored industrial waste as a solid precursor (40% red mud). This might reduce the environmental impact associated with the waste’s current management strategy.

## Figures and Tables

**Table 1:** Characterization of surfactants.

ID	Chemical Formula	Type	Producer
Hostapur OSB	Olefin sulphonate	Anionic	Clariant
SDS	Sodium dodecyl sulfate	Anionic	Sigma-Aldrich
Triton^®^ X-114	Nonylphenol-polyethylene glycol	Non-ionic	Acros Organics

**Table 2:** Oxide composition of RM and MK.

Oxides Present (wt.%)	RM	MK
Fe_2_O_3_	48.4	1.85
Al_2_O_3_	19.2	40.1
SiO_2_	6.30	52.1
TiO_2_	6.67	1.97
Na_2_O	5.77	0.08
CaO	1.10	0.13
P_2_O_5_	0.36	0.07
SO_3_	0.22	0.04
MgO	0.07	0.27
K_2_O	0.07	1.09
Loss on ignition (L.O.I.)	11.0	2.24

**Table 3:** Geometric density, porosity, thermal conductivity, and water absorption values of GFs.

Surfactant Type	Content of Surfactant/Al (wt.%)		Geometric Density (kg/m^3^)	Total Porosity (%) (by He- Pycnometer)	Open Porosity (%) (by Archimedes)	Closed Porosity (%)	Thermal Conductivity (W/m.K)	Water Absorption (%)
OSB	0.025	0.05	580 ± 22	73.8 ± 0.9	39.3 ± 0.7	34.5	0.127 ± 0.004	64.0 ± 0.4
	0.05		570 ± 65	74.4 ± 2.9	40.5 ± 1.3	33.9	0.121 ± 0.003	63.9 ± 3.6
	0.075		560 ± 28	74.9 ± 1.2	40.4 ± 1.0	34.5	0.129 ± 0.003	64.2 ± 3.5
	0.025	0.075	480 ± 20	78.5 ± 0.9	45.1 ± 0.9	33.4	0.119 ± 0.003	80.4 ± 2.5
	0.05		500 ± 13	77.0 ± 0.6	46.5 ± 0.5	30.5	0.107 ± 0.003	72.9 ± 1.4
	0.075		450 ± 39	78.0 ± 1.7	45.0 ± 1.7	33.0	0.091 ± 0.001	73.2 ± 5.2
	0.025	0.1	430 ± 16	80.4 ± 0.7	44.0 ± 0.3	36.4	0.120 ± 0.002	74.8 ± 4.3
	0.05		460 ± 13	79.2 ± 0.5	47.7 ± 0.7	31.5	0.100 ± 0.005	77.4 ± 1.5
	0.075		510 ± 18	76.9 ± 0.8	45.0 ± 0.3	31.9	0.104 ± 0.005	73.9 ± 1.1
SDS	0.025	0.05	590 ± 20	73.6 ± 0.8	28.3 + 0.5	45.3	0.129 ± 0.006	42.2 ± 0.7
	0.05		560 ± 12	74.6 ± 0.5	28.9 + 0.5	45.7	0.126 ± 0.003	43.9 ± 1.2
	0.075		540 ± 13	75.6 ± 0.5	28.8 + 3.7	46.8	0.126 ± 0.005	44.5 ± 1.3
	0.025	0.075	470 ± 28	78.8 ± 1.2	32.7 + 0.9	46.1	0.115 ± 0.005	51.4 ± 1.2
	0.05		520 ± 18	76.7 ± 0.8	30.6 + 5.8	46.1	0.117 ± 0.002	48.6 ± 1.3
	0.075		460 ± 10	79.2 ± 0.4	30.6 + 0.9	48.6	0.108 ± 0.005	52.3 ± 0.5
	0.025	0.1	400 ± 12	82.2 ± 0.5	32.3 + 1.8	49.9	0.113 ± 0.002	58.0 ± 2.4
	0.05		410 ± 10	81.6 ± 0.4	41.2 + 0.7	40.4	0.090 ± 0.002	72.1 ± 4.6
	0.075		410 ± 3	81.4 ± 0.1	40.7 + 1.1	40.7	0.103 ± 0.001	72.1 ± 1.7
X114	0.025	0.05	580 ± 21	73.8 ± 0.9	36.5 ± 0.7	37.3	0.126 ± 0.007	53.3 ± 1.3
	0.05		630 ± 38	71.6 ± 1.7	39.9 ± 2.8	31.7	0.126 ± 0.002	53.0 ± 1.3
	0.075		580 ± 29	73.7 ± 1.3	39.3 ± 0.4	34.4	0.107 ± 0.005	56.6 ± 2.0
	0.025	0.075	540 ± 41	75.5 ± 1.8	39.3 ± 0.7	36.2	0.121 ± 0.006	57.5 ± 0.1
	0.05		510 ± 30	77.1 ± 1.3	40.8 ± 1.3	36.3	0.116 ± 0.003	64.5 ± 0.8
	0.075		580 ± 24	73.7 ± 1.0	38.4 ± 0.1	35.3	0.102 ± 0.006	57.0 ± 1.4
	0.025	0.1	530 ± 12	76.1 ± 0.5	41.3 ± 1.0	34.8	0.115 ± 0.002	60.5 ± 1.7
	0.05		490 ± 10	77.8 ± 0.4	40.2 ± 0.6	37.6	0.102 ± 0.004	62.7 ± 1.0
	0.075		564 ± 54	74.6 ± 2.4	40.9 ± 0.5	33.7	0.094 ± 0.007	56.9 ± 3.2

## Data Availability

Not applicable.
